# The relationship between nursing home quality and costs: Evidence from the VA

**DOI:** 10.1371/journal.pone.0203764

**Published:** 2018-09-19

**Authors:** Kathleen Carey, Shibei Zhao, A. Lynn Snow, Christine W. Hartmann

**Affiliations:** 1 Department of Health Law, Policy and Management, Boston University School of Public Health, Boston, Massachusetts, United States of America; 2 Center for Healthcare Organization and Implementation Research, Edith Nourse Rogers Memorial Veterans Hospital, Bedford, Massachusetts, United States of America; 3 Tuscaloosa Veterans Affairs Medical Center, Tuscaloosa, Alabama, United States of America; 4 Alabama Research Institute on Aging and the Department of Psychology, University of Alabama, Tuscaloosa, Alabama, United States of America; West Chester University of Pennsylvania, UNITED STATES

## Abstract

Ensuring quality of care in nursing homes is a public health priority, yet how nursing home quality relates to cost is not well understood. This paper addresses this relationship for 132 VA community living centers (nursing homes), for fiscal years 2014 and 2015. We estimated cost models using the VA Decision Support System which tracks total direct costs and nursing direct costs for individual resident segments of care. We summed residents’ total costs and nursing costs to the community living center level for each year. Annual facility costs then were regressed on quality of care measured with composite scores based on 13 distinct adverse events. Results indicated that higher quality was associated with higher predicted cost. However, we did not find evidence that higher costs were driven by high nurse staffing levels.

## Introduction

The policy challenge of controlling U.S. health care spending elevates the importance of understanding the relationship between health care quality and cost, an issue particularly relevant in nursing homes, which have long battled poor quality [1–3]. Ostensibly, if quality improvements involve added expense, it follows that higher health care quality would be associated with higher cost. However, poor quality due to lapses in provider care can trigger adverse events such as infections or falls, requiring additional resources to repair damages. Despite long-standing interest in this issue, a recent systematic review concluded that the association between health care quality and cost is inconsistent with moderate effects in either direction, recommending that future studies focus on identifying which types of spending are most effective in improving quality of care [4].

A substantial body of literature has addressed the quality cost relationship in the context of hospital care, and several studies have analyzed the issue in U.S. nursing homes. The majority of nursing home studies examined the association between budget allocation or accounting costs and various health outcome measures such as pressure ulcers [5–8], decline in activities of daily living [5–6] weight loss [5], cognitive and mood decline [7], process measures such as use of physical restraints, urethral catheters or feeding tubes [8], or a composite of such measures [9]. Other studies examined the relationship between Medicaid payments and nursing quality using structure [10], process [11], or outcome measures [12]. Overall, while some of these studies showed a positive relationship between quality and cost or payments, the majority produced mixed results or were indeterminate.

The Veterans Health Administration (VA) is a federal, globally-budgeted health care system that operates 135 community living centers (CLCs; formerly called nursing homes) across the U.S. VA is a good setting for examining the association between nursing home quality and costs because a key challenge in understanding the relationship between health care quality and cost is effectively measuring patient costs. VA has a comprehensive and generally unparalleled system of capturing cost; few health care provider information systems have similarly good measures of cost at the level of a unique patient stay [13]. VA measures actual resources used in a specific patient or resident stay, which provides detailed information not captured in CLC budget allocations, which are based on a capitated system. VA also collects comprehensive data on CLC quality using the standard measures compiled by the Centers for Medicare and Medicaid Services (CMS) for skilled nursing facilities servicing Medicare beneficiaries [14].

This study examined the association between CLC quality and cost with an econometric analysis of CLC facility-level data for fiscal years 2014 and 2015 (FY14 and FY15). The main finding was that higher quality as gauged by better outcomes was associated with higher CLC costs.

## Data

### Resident assessment instrument

The Resident Assessment Instrument (RAI) is an international standard assessment tool used for determining the care requirements of nursing home residents. A core component of the RAI is the Minimum Data Set (MDS), a data collection tool consisting of approximately 300 items that summarize individual residents’ clinical status and functional capabilities. Residents are categorized according to MDS data into Resource Utilization Groups (RUGs), a classification system that provides a foundation for formulating individual care plans. RAI/MDS Version 3.0, including RUG Version IV, is part of the U.S. federally mandated process for clinical assessment of all residents of nursing homes certified to participate in Medicare or Medicaid and is used by VA for assessment of CLC residents [15].

VA generates a set of MDS-based quality measures (QMs) using metrics developed by CMS for purposes of monitoring and promoting quality improvement and facilitating nursing home choice. We obtained CLC-level data on 13 VA adverse event QMs calculated for residents in 132 CLCs during FY14 and in FY15. The QMs are expressed as rates, in which the numerator captures the number of adverse events and the denominator contains the number of residents eligible for that event. Assessments are conducted at multiple times during the year. Table 1 describes the QMs and lists the CLC level mean values of the number of residents eligible for each QM (denominator) and the number of events (numerator) by year. To illustrate for Falls with Major Injury in FY14, there were 643 resident assessments for which this adverse event might have occurred. Of those, 15 residents experienced the event, indicating a rate of 2.3 percent.

**Table 1:** CLC mean values of quality monitor resident eligibility and adverse events.

Measure	FY14		FY15	
	Number of resident assessments	Number of adverse events	Number of resident assessments	Number of adverse events
Falls with Major Injury	643	15	630	15
Reports Moderate to Severe Pain	430	113	430	112
High-Risk Residents with Pressure Ulcers	353	37	348	35
Residents with Urinary Tract Infection	636	67	625	60
Low-Risk Residents Who Lose Control of B/B^†^	267	68	269	70
Catheter Inserted and Left in Bladder	563	59	559	59
Need for Help with ADLs^‡^ Increased	493	98	483	93
Residents Who Lose Too Much Weight	622	69	609	66
Residents with Depressive Symptoms	565	32	550	30
Received Antipsychotic Medication	613	189	518	112
Residents Who Had a Fall	643	266	630	259
Prevalence of Antianxiety or Hypnotic Medication	330	51	322	43
Behavior Symptoms Affecting Others	558	177	543	169

^†^Bowel/Bladder;

^‡^Activities of Daily Living

### Decision support system

We captured resident-level RUG and cost data using the VA Decision Support System (DSS). The RUG-IV classification system has eight major categories: Rehabilitation plus Extensive Services, Rehabilitation, Extensive Services, Special Care High, Special Care Low, Clinically Complex, Behavioral Symptoms and Cognitive Performance Problems, and Reduced Physical Function. These are further divided into 66 RUG groups according to factors including intensity of the resident’s activities of daily living (ADL) needs, the presence of depression, and the provision of restorative nursing services. DSS divides all CLC encounters for a given fiscal year into segments, or time periods comprising contiguous days during which a resident is assigned to a specific RUG group, such that a change in RUG assignment generates a new segment of care. DSS data include the RUG group assigned to each segment and the number of days in the segment.

To capture costs, DSS applies activity-based costing (ABC), a bottom-up approach that sums the cost of intermediate products and services provided during a resident stay. ABC systems are considered to be the best estimates of the true economic costs of the production of health services [16]. DSS disaggregates the total cost of each segment of care into direct (resident cost) and indirect (overhead and administration cost) components. Direct resident costs are further disaggregated into fixed and variable components according to whether or not they vary with volume of services. DSS also distributes total, direct, and indirect costs according to six service categories: nursing & residential care, radiology, surgery, pharmacy, laboratory, and all other.

## Methods

### Variables

We selected two dependent cost variables to correspond with the components of cost most immediately related to direct resident care. First is total variable direct costs (TOTVD) which excludes indirect (overhead and administrative) and other fixed costs. Because opportunities for avoiding adverse resident events fall primarily to nursing staff, we also selected the subcomponent of TOTVD that is distributed to nursing & residential care (NSGVD). We summed these variables across segments to obtain facility level totals for each fiscal year.

For the key independent variable, we measured quality in two ways. First, we drew on recent literature describing methods of combining individual quality indicators into composite measures [17], an approach that increases precision of estimates particularly in small samples [18] and that has been used in previous study of VA nursing homes [19–20]. Specifically, we calculated opportunity-based weights by summing the number of adverse events across types of events and dividing by the sum of the number eligible for each event (reflecting the opportunity for an adverse event to occur). Opportunity-based weights have the advantage of taking account of the different types of residents that the CLC might care for. Second, we entered quality as the sum of adverse events. This approach has not, to our knowledge, been used in nursing home studies before. It has, however, been used in a facility-level study of the relationship between costs and quality in hospitals that summed adverse events as captured in the Patient Safety Indicators constructed by the Agency for Healthcare Research and Quality [21]. Results from that study showed superior performance from a model which used a summed adverse event quality measure compared to a model which used individual adverse event rates. This study extends use of this approach to study of nursing home costs [22].

The models also incorporated three covariates. First is the number of admissions. We obtained these by combining sequential segments by unique resident to identify resident stays. We defined admissions as the sum of the number of resident stays for each CLC in each year. A long-term resident whose stay spanned both fiscal years was counted as an admission in each year. To control costs for length of time residents were in care, we included average length of stay as a second cost driver.

Finally, following previous VA research on CLCs, we adjusted for resident acuity using the 66 RUG group case-mix values [14]. To construct a facility level case-mix we first determined the distribution of facilities’ total resident days according to the number of days assigned to each RUG group. We used this distribution to produce weights corresponding to each RUG group’s percentage of total resident days. We then calculated a weighted average of the RUG case-mix values for each CLC in each year. The RUG group case-mix values vary according to whether a nursing home is located in a rural or urban area. We applied the rural and urban variants in our facility-level construct according to the location of the CLC.

Table 2 displays descriptive statistics for the 264 observations obtained from two years of annual data on the 132 CLCs (i.e., one observation per CLC per year). On average, total variable direct costs were $7.381 million; the nursing component accounted for 56% of these, or $4,148 million. The mean number of CLC admissions was 293, averaging 161 days of residence per admission. The mean value of the composite quality monitor across observations was 17.7% and the mean value of the sum of adverse events was 1,183.

**Table 2:** Dependent and independent variable descriptive statistics: CLCs for 2014–2015.

Variable	Mean	Standard Deviation	Minimum	25^th^ Percentile	50^th^ Percentile	75^th^ Percentile	Maximum
**Dependent Variables**							
Total Variable Direct Costs (thousand $)	7,381	5,462	420	3,715	5,952	9,637	35,207
Nursing Variable Direct Costs (thousand $)	4,148	3,248	245	1,973	3,350	5,455	22,947
**Independent Variables**							
Number of Admissions	293	192	13	157	246	377	1,144
Average Length of Stay	161	115	23	67	125	233	365
Case-Mix Index	17.9	3.73	5.44	15.7	17.7	20.2	17.0
Quality Composite	0.177	0.037	0.054	0.151	0.177	0.203	0.286
Number of Adverse Events	1,183	928	17	485	922	1,768	5,008
Number of CLCs Over Two Years	264						

#### Statistical analysis

Following previous literature modeling hospital cost as a function of output and quality constructed from adverse event metrics [19], we specify facility cost as

$$C=e^{f+u}\to \text{ln}TC=f+u$$ (1)

where

$$f=\alpha +\beta_{1\;}Admissions+\beta_{2}\;LOS+\beta_{3}\;Casemix+\beta_{4}\;Quality$$

and where

*C* = total variable direct costs, nursing variable direct costs

*Admissions* = number of unique resident admissions

*LOS* = average number of resident days per admission

*Casemix* = weighted average of RUG case-mix values

*Quality* = composite QM rates, summed QM events

We estimated four variants of (1), accounting for two measure of the dependent variable and two alternative approaches to measuring quality.

Generalized linear modeling (GLM) estimation techniques are frequently used in models of patient costs, which tend to exhibit significant positive skewness. Because our model sums individual resident costs to the facility level, we conducted a preliminary test to determine whether GLM was appropriate for estimation of Eq (1). Ordinary least squares (OLS) estimation is frequently preferable to GLM when the log scale residuals obtained from OLS estimation are heavy-tailed. (kurtosis > 3) [23]. Accordingly, we examined the residuals obtained from four OLS regression for Eq (1). Because the estimated values of kurtosis fell in the range of 1.37–1.73, we proceeded with GLM [24]. Based on application of the modified Park test [23, 25–26], which assumes that the conditional variance follows a power relationship to the mean, we assumed an underlying gamma distribution.

The data contain annual observations over a two-year period. To account for correlation among observations on individual CLCs over time, we fit a generalized estimating equation (GEE) model [27]. GEE is a GLM approach to repeated measures that is preferable to fixed effects when interest is in population averaged rather than individual effects. We estimated the model using PROC GENMOD, SAS Version 9.2, with the REPEATED option, which invokes GEE.

## Results

Table 3 presents results of the GLM estimation of total variable direct costs on workload and quality measures. The first model enters the quality monitors as a composite measure of the rate of occurrence. In this model, admissions and length of stay are highly significant in explaining cost variation, as expected. The negative sign on quality, which is weakly significant, indicates that, on net, poorer quality was associated with lower costs. The results of the second model, which enters the quality monitors as summed events, exhibit similar results for the workload variables, as indicated by coefficients that differ little from the composite measure model. The coefficient on quality is also negative and moderately significant in the summed events model.

**Table 3:** Generalized linear model estimates of total variable direct costs.

Variable	Composite Measure of Quality		Summed Events Measure of Quality	
	Coefficient	Standard Error	Coefficient	Standard Error
Log of Number of Admissions	0.982***	0.047	1.034***	0.050
Log of Average Length of Stay	0.326***	0.045	0.382***	0.061
Case-Mix Index	-0.016	0.010	-0.019*	0.011
Quality Monitors: Composite	-1.28*	0.679	---	---
Quality Monitors: Summed Events	---	---	-0.083 E-3**	0.042 E-3
Intercept	9.195***	0.586	8.577***	0.560
Number of Observations	264			

* p < 0.10;

**p < 0.05;

***p < 0.01

Table 4 indicates the results of the GLM estimation of the nursing component of variable direct costs. In both models, variation in workload is again significantly associated with variation in costs, as anticipated. The coefficients on the quality measures, however, while exhibiting negative signs, are not statistically different from zero.

**Table 4:** Generalized linear model estimates of nursing variable costs.

Variable	Composite Measure of Quality		Summed Events Measure of Quality	
	Coefficient	Standard Error	Coefficient	Standard Error
Log of Number of Admissions	1.008***	0.053	1.057***	0.050
Log of Average Length of Stay	0.393***	0.051	0.449***	0.067
Case-Mix Index	-0.022*	0.013	-0.024*	0.013
Quality Monitors: Composite	-0.725	0.779	---	---
Quality Monitors: Summed Events (100)	---	---	-0.075 E-3	0.047 E-3
Intercept	8.156***	0.687	7.623***	0.609
Number of Observations	264			

* p < 0.10;

***p < 0.01

Finally, in Tables 5 and 6, we examine the coefficients on the quality measures more closely. To provide a useful interpretation, we calculated cost predictions from both models, allowing the quality measures to vary across the interquartile range observed in the data. Table 5 shows a fall in the composite measure model from $7.524 million at the 1^st^ quartile value of the quality composite (15.1%) to $7.039 million at the 3^rd^ quartile (17.7%), a 6.4% difference. For nursing costs, the difference is smaller, 3.7% ($4.203 million compared to $4.047 million). The summed events measure model cost predictions shown in Table 6 are higher than the composite measure model prediction in both absolute and relative terms: $7.969 million to $7.164 million (a 10.1% difference) for total cost and $4.489 million to $4.077 million (a 9.2% difference) for nursing costs.

**Table 5:** Cost predictions at quartile measures of quality: Composite measure of quality.

Percentile	Quality: Composite	Predicted Cost (000 $)	
		Total Cost	Nursing Cost
**25**^**th**^	0.151	7,524	4,203
**50**^**th**^ **(Median)**	0.293	7,278	4,124
**75**^**th**^	0.177	7,039	4,047

**Table 6:** Cost predictions at quartile measures of quality: Summed events measure of quality.

Percentile	Quality: Number of Summed Events	Predicted Cost (000 $)	
		Total Cost	Nursing Cost
**25**^**th**^	485	7,969	4,489
**50**^**th**^ **(Median)**	922	7,686	4,344
**75**^**th**^	1,768	7,164	4,077

## Discussion

The hypothetical relationship between health care quality and cost supports two theoretical interpretations. From an economic perspective, if greater resources are allocated to improve structural elements of quality, higher costs may result [21]. Alternatively, in the quality management view, high quality health care organizations can reduce costs by investing resources in preventive steps that can lead to lower amounts of waste in the production process [8, 28]. This study sought to clarify this relationship in the context of 132 VA CLCs. Using two different metrics based on 13 outcome measures of quality, we found that higher level of quality was associated with higher predicted cost, consistent with the economic interpretation. This finding contrasts with a patient-level study of the relationship between quality and costs in VA inpatient settings. That study, which also used the VA DSS costing system and outcome measures of quality, found higher rates of adverse patient safety events to be associated with higher patient costs [21].

It is outside the scope of this study to draw conclusions about specific cost drivers in VA CLCs related to outcome measures of quality. However, a few salient points arise. In examining nursing costs only, while quality and cost still moved in the same direction, the association was weaker and statistically insignificant. This suggests that higher costs are not being driven mainly by higher structural quality related to nurse staffing levels. It may be that CLCs with lower overall cost are not performing as well as higher cost CLCs, suggesting possible reverse causality. Another possible contributor to the positive association between quality and cost is unmeasured case mix. Residents who are in poorer health may be more likely to have a fall, pressure ulcers, or receive antipsychotic medication, for example, and may also be relatively high cost residents.

There are limitations to this study and results should be interpreted with caution. Because information on the number of adverse events was available only at the CLC level, we conducted the analysis at the facility level. This resulted in loss of information on patient costs, which precluded our ability to identify an association between quality and cost as it relates to patient factors. Additionally, while combining individual outcome measures to single measures was applicable in this dataset, it is possible that the direction of association varies across measures and that what we are observing is a net effect, albeit of moderate magnitude. Combining events may be particularly salient for nursing costs, because events vary according to intensity of nursing services. For example, pressure ulcers and ADL change are clearly nursing sensitive while receipt of medication may be more reflective of the roles of activity staff or pharmacists. Moreover, this is a retrospective cross-sectional study and, as such, can only report observed associations. What we can reasonably conclude is that there was no evidence that lapses in quality of care in VA CLCs led to higher occurrence of cost-increasing adverse events.

Future study of VA CLC costs can clarify these issues further. Longitudinal data at the facility level could be instrumental in improving the capacity for identifying causal effects. A second useful direction for research would be to obtain resident level information on quality that could be aligned with DSS resident level costs. Better understanding of how quality and cost in VA CLCs are interrelated will have value for management of VA CLCs, in addition to being informative in non-VA settings that lack good measures of resident-level costs.
